# Application of a humeral intramedullary nail for subtrochanteric fracture in a patient with poliomyelitis sequelae: a case report

**DOI:** 10.3389/fsurg.2026.1788560

**Published:** 2026-02-26

**Authors:** Xiang Yu, Wen-Bo Sheng, Rong-Guang Ao, Bing-Li Liu, Jun Zhang

**Affiliations:** Department of Orthopedics, The Seventh People’s Hospital Affiliated to Shanghai University of Traditional Chinese Medicine, Shanghai, China

**Keywords:** case report, fracture fixation, humeral intramedullary nail, poliomyelitis sequelae, subtrochanteric fracture

## Abstract

**Objective:**

To investigate the clinical feasibility and efficacy of using a humeral intramedullary nail for the treatment of subtrochanteric fracture in a patient with ipsilateral poliomyelitis sequelae.

**Methods:**

A case of a 54-year-old female patient with a left subtrochanteric fracture caused by a traffic accident was reported. The patient had a 50-year history of ipsilateral poliomyelitis, leading to developmental deformity and severe stenosis of the femoral medullary canal in the affected limb, which could not accommodate a conventional femoral intramedullary nail. Therefore, we innovatively used a 7 mm diameter humeral interlocking intramedullary nail for internal fixation. Limited open reduction was performed during surgery, supplemented with cerclage wiring to enhance fracture stability. The medullary canal was reamed to 8 mm, after which the nail was successfully inserted.

**Results:**

The surgical procedure was smooth. Intraoperative fluoroscopy and postoperative x-rays showed satisfactory fracture reduction and good positioning of the internal fixation. The patient was followed up for 30 months. Imaging examinations confirmed bony union of the fracture without complications such as failure of internal fixation. Ultimately, the walking and flexion functions of the left lower limb recovered to the pre-injury level.

**Conclusion:**

For special types of subtrochanteric fractures with severe femoral medullary canal stenosis due to conditions like poliomyelitis sequelae, the application of a humeral intramedullary nail is a safe and effective innovative treatment strategy. This approach provides reliable intramedullary fixation. This experience offers a valuable reference for managing similar complex orthopedic problems.

## Introduction

The treatment of subtrochanteric fractures is particularly challenging when combined with poliomyelitis sequelae, as the affected limb often presents anatomical abnormalities such as severe medullary canal stenosis, osteoporosis, and deformity, making standard femoral intramedullary nails inapplicable. To address this challenge, this study reports a case of a 54-year-old female with ipsilateral poliomyelitis sequelae and a subtrochanteric fracture, where a thinner humeral intramedullary nail was innovatively used for internal fixation. By detailing the preoperative planning, surgical technique, and follow-up results, this study aims to explore the feasibility and efficacy of this alternative method for special types of subtrochanteric fractures, providing a reference for the clinical management of similar complex situations.

## Case presentation

### Chief complaints

Left hip pain and limited mobility for 2 h after a traffic accident.

### History of present illness

The patient, a 54-year-old female, was injured in a traffic accident while cycling 2 h prior, resulting in left hip pain and limited mobility. She was sent to our emergency department, where x-rays indicated a “left subtrochanteric fracture”. She was admitted for further treatment.

### History of past illness

The patient has a 50-year history of poliomyelitis.

### Personal and family history

The patient declared no family history of genetic diseases.

### Physical examination

The patient was lying supine with a pained expression. Generalized muscle atrophy was observed in the left lower limb, with surgical scars visible on the left proximal thigh and knee. Significant swelling and subcutaneous ecchymosis were present in the left hip and upper thigh, with the limb exhibiting shortening and external rotation deformity. Obvious tenderness was noted in the left upper thigh, with palpable bone crepitus and abnormal movement; axial percussion pain was positive. Due to pain protection, all ranges of motion in the affected limb were severely limited, and muscle strength could not be assessed cooperatively. Dorsalis pedis artery pulses were palpable bilaterally, with normal capillary refill time; active toe movement was present, and no significant decrease in superficial sensation on the dorsum and sole of the foot was noted. Limb length measurement indicated structural shortening of the left lower limb.

### Laboratory examinations

The patient's vital signs were as follows: temperature, 37.2 °C; pulse rate, 102 beats/min; respiration rate, 35 breaths/min; and blood pressure, 112/52 mmHg. A blood test revealed that the white blood cell count was 9.6 × 109/L, the neutrophil count was 71%, the haemoglobin level was 101 g/L, and the C-reactive protein level was 22 mg/L. The rest of the laboratory tests were unremarkable.

### Imaging examinations

**Figure 1:**
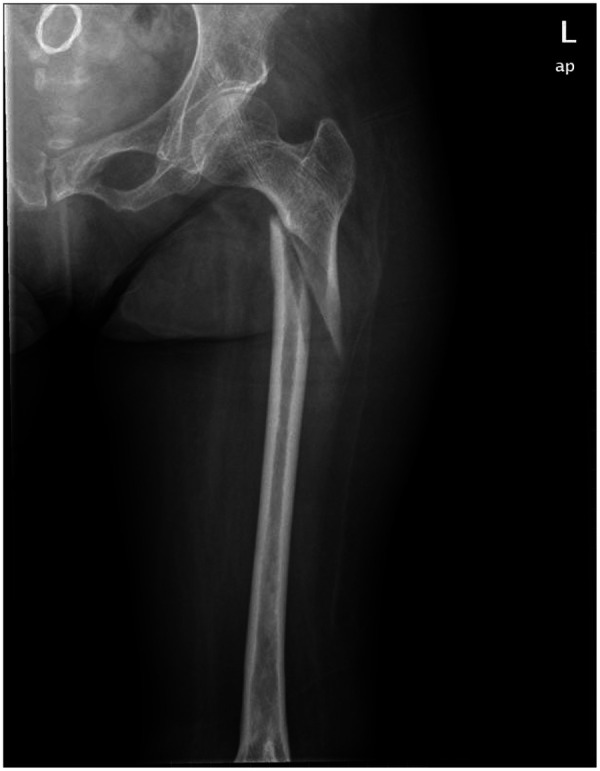
Preoperative anteroposterior x-ray of the left hip showed a left subtrochanteric femoral fracture AO/OTA type A3.

x-ray showed a left subtrochanteric fracture (Figure 1).

### Final diagnosis

1. Left subtrochanteric fracture, AO/OTA A3 type.
2. Poliomyelitis sequelae.

### Treatment

#### Perioperative management

A comprehensive perioperative management protocol was implemented to ensure patient safety and optimize outcomes. Upon admission, the patient's general condition was thoroughly evaluated. Given the trauma and pre-existing disability, nutritional status was assessed, and nutritional support was initiated to address the mild anemia (haemoglobin 101 g/L). Multimodal analgesia was employed to effectively control pain. Pharmacological thromboprophylaxis with low-molecular-weight heparin was administered preoperatively and continued postoperatively, considering the high risk of deep vein thrombosis associated with lower limb fractures and anticipated reduced mobility. The anesthesia team conducted a preoperative assessment, and general anesthesia was selected. Intraoperative measures included antibiotic prophylaxis administered 30 min before incision and careful monitoring of hemodynamic status and fluid balance. This holistic approach aimed to mitigate systemic risks and create optimal conditions for surgery and recovery.

#### Preoperative preparation

Following the general perioperative optimization, the specific surgical plan was determined to be open reduction and internal fixation with an intramedullary nail. Preoperative measurements revealed a critical anatomical constraint: the narrowest diameter of the femoral medullary canal was only 4.5 mm, rendering commonly used femoral intramedullary nails unsuitable. Consequently, a humeral intramedullary nail (Smith & Nephew, TRIGEN, proximal bend humeral long nail, 8/7 mm, 28 cm) was selected as a viable alternative to achieve stable intramedullary fixation. Although a plate-screw system was considered as it could circumvent the issue of medullary canal stenosis, this option was ultimately rejected. The decision was based on the high risk of mechanical failure (such as screw loosening or plate breakage) associated with plates due to stress concentration in the high-stress subtrochanteric region, a risk exacerbated by the patient's osteoporotic bone quality resulting from long-term disuse.

#### Surgical procedure

**Figure 2:**
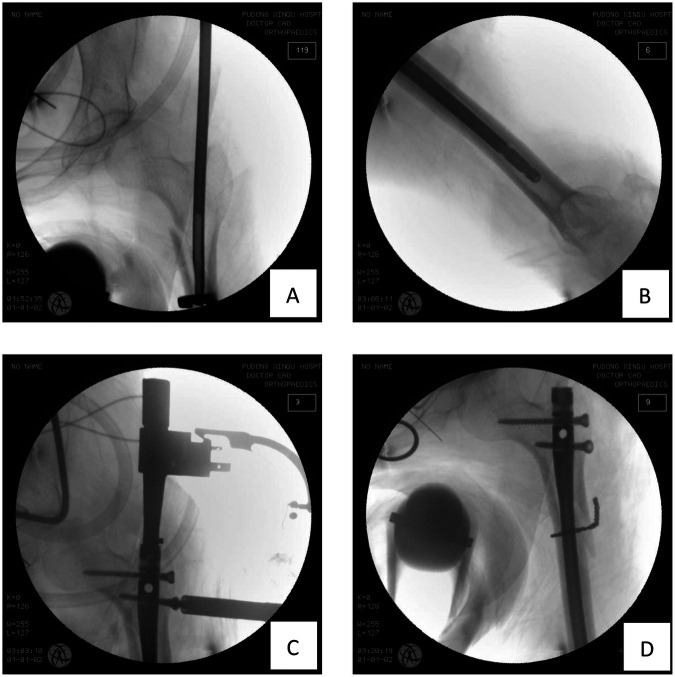
Intraoperative imaging: **(A)** insertion of intramedullary nail. **(B)** The intramedullary nail is of appropriate length and well-matched in diameter. **(C)** Insert the proximal locking screw. **(D)** Intraoperative fluoroscopy showed satisfactory positioning of the intramedullary nail and wires with good fracture reduction.

After satisfactory anesthesia, the patient was placed in the supine position. Routine disinfection and draping were performed. A straight lateral incision approximately 15 cm long was made over the left hip, and layers were incised to expose the tip of the greater trochanter and the subtrochanteric fracture line. Under direct vision, the fracture was reduced using reduction forceps and then stabilized with cerclage wiring. The entry point was selected slightly medial to the tip of the greater trochanter. A guide wire was inserted, and its position within the medullary canal was confirmed as satisfactory by C-arm fluoroscopy. Subsequently, flexible reamers were used starting from 5 mm, progressively reaming the medullary canal until the proximal femoral canal was enlarged to 8 mm, with the cortical bone remaining intact. A 7 mm diameter, 28 cm long humeral interlocking intramedullary nail was selected and smoothly inserted along the guide wire into the medullary canal. Fluoroscopy confirmed good fracture alignment and appropriate depth of the nail. The proximal aiming device was installed, and two proximal locking screws were successfully inserted. Distal locking was not performed. Repeat anteroposterior and lateral fluoroscopy confirmed satisfactory fracture reduction, good position of the intramedullary nail and proximal locking screws, and stability of the fracture site. The surgical field was irrigated, hemostasis was thoroughly achieved, and the incision was closed in layers. The surgery proceeded smoothly with an estimated blood loss of about 200 mL; no blood transfusion was required, and the patient returned to the ward safely after surgery (Figure 2).

#### Postoperative rehabilitation and follow-up

x-ray review three days postoperatively showed satisfactory fracture reduction and well-matched internal fixation.

The postoperative rehabilitation plan followed the principles of “individualization, safety, and progression”, tailored according to the patient's ipsilateral limb muscle weakness, slender bone structure, and internal fixation characteristics. In the early postoperative period (0–4 weeks), the focus was on bed rest with strict non-weight-bearing, emphasizing ankle pump exercises, isometric contractions of the affected limb muscles, and passive range of motion exercises for the hip and knee joints to reduce swelling, alleviate pain, and prevent complications. In the mid-term (starting 5–8 weeks postoperatively), after x-ray confirmation of callus formation, gradual partial weight-bearing with the assistance of crutches was initiated, along with enhanced range of motion and muscle strength training for the hip and knee. In the later stage (approximately 3 months postoperatively), based on fracture healing progress, a gradual transition to full weight-bearing was made, focusing on strengthening stabilizing muscle groups like the gluteus medius, gait training, and functional exercises to restore daily activity capacity. Fall prevention was strictly emphasized throughout the process. Regular follow-up examinations were conducted, and the rehabilitation progress was dynamically adjusted based on imaging results and functional recovery.

**Figure 3:**
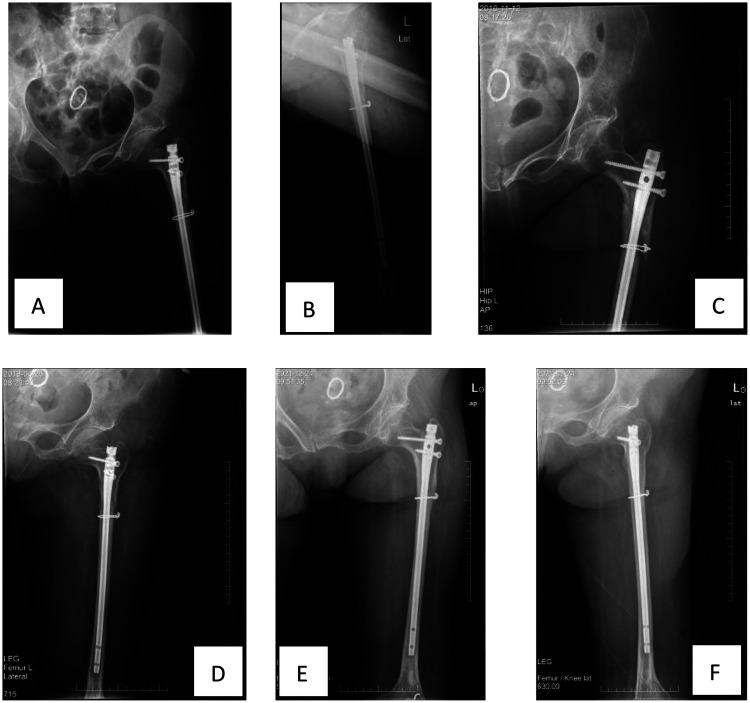
Postoperative follow-up: **(A)** anteroposterior x-ray on the third postoperative day. **(B)** Lateral x-ray film 3 days postoperatively. **(C)** Anteroposterior x-ray at 1 month postoperatively. **(D)** Anteroposterior x-ray at 5 months postoperatively. **(E)** Anteroposterior x-ray at 30 months postoperatively. F. Lateral x-ray at 30 months postoperatively.

**Figure 4:**
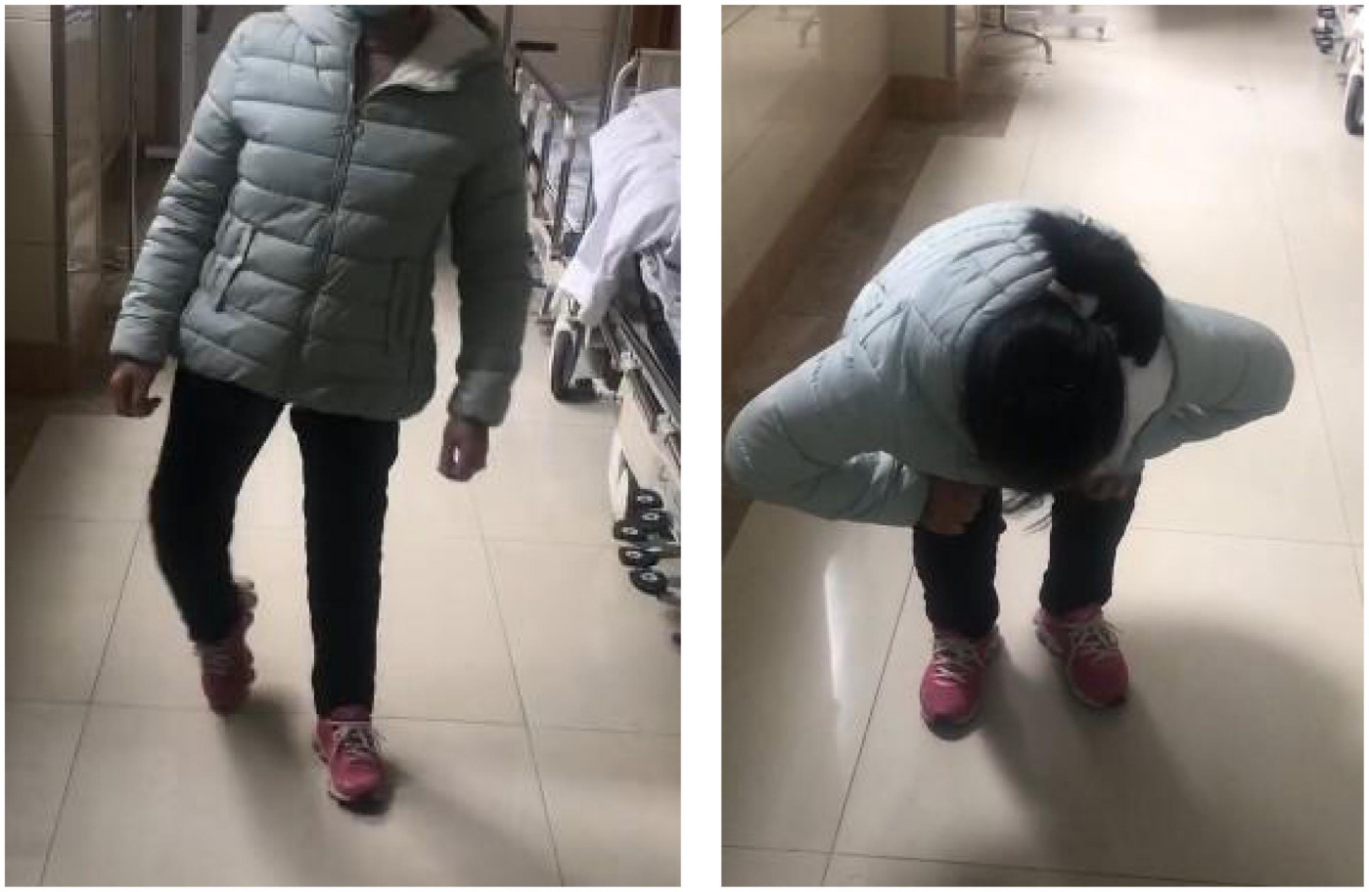
At 30 months post-operation, the patient's walking and squatting functions have recovered to the pre-injury state.

The patient returned for follow-up examinations at 1 month, 5 months, and 30 months postoperatively. Follow-up x-rays showed good fracture recovery and satisfactory position of the internal fixation (Figure 3). The patient essentially recovered limb function to the pre-injury level (Figure 4).

#### Timeline

See Table 1.

**Table 1:** Timeline.

Timepoint	Key event	Main findings and decisions
Day of Injury	Traffic accident injury, Emergency visit	x-ray confirmed left subtrochanteric fracture (AO/OTA A3 type)
Preoperative Preparation	Post-admission completion of examinations	Imaging measurements revealed medullary canal stenosis (narrowest 4.5 mm), decision to use humeral intramedullary nail
Surgery Day	Open reduction and internal fixation surgery	Successful insertion of 7 mm humeral intramedullary nail, assisted by cerclage wiring, no distal locking performed
Postoperative Day 3	First postoperative x-ray review	Confirmed good position of internal fixation, satisfactory fracture reduction
Postoperative 1 Month	Outpatient follow-up	x-ray showed early callus formation, guidance for non-weight-bearing rehabilitation
Postoperative 5 Months	Outpatient follow-up	x-ray showed blurred fracture line, continuous callus, guidance for gradual weight-bearing
Postoperative 30 Months	Outpatient follow-up	Clinical assessment indicated recovery of walking and squatting functions to pre-injury leve

## Discussion

This case report presents a challenging case of subtrochanteric fracture, its particularity lying in the patient's concomitant ipsilateral limb poliomyelitis sequelae. The core challenge and innovation of this case lie in the successful application of a humeral intramedullary nail to address the internal fixation problem posed by femoral developmental deformity and severe medullary canal stenosis resulting from poliomyelitis sequelae.

Poliomyelitis sequelae lead to muscle atrophy, muscle imbalance, and long-term disuse in the affected limb, subsequently causing skeletal hypoplasia, manifesting as a slender femur, thin cortex, and narrow medullary canal (1). These anatomical abnormalities make patients more susceptible to fractures during daily activities, and once a fracture occurs, its internal fixation treatment faces significant challenges (2). In this case, preoperative measurement revealed the narrowest diameter of the femoral medullary canal was only 4.5 mm, far below the minimum diameter required for conventional proximal femoral intramedullary nails. This anatomical abnormality rendered standard femoral intramedullary nail systems completely inapplicable. While plate and screw systems could avoid the medullary canal issue, their biomechanical disadvantages pose a high risk of failure for extramedullary fixation in the high-stress subtrochanteric region (3). Plate fixation, as a load-bearing device, is susceptible to failure due to stress concentration at the screw-bone interface, particularly in osteoporotic bone. Given the patient's long-term limb disuse and associated osteopenia secondary to poliomyelitis, the risk of implant failure with a plate construct was deemed unacceptably high. Therefore, selecting a device that can both adapt to the narrow medullary canal and provide strong intramedullary fixation is crucial for such patients.

In this case, we innovatively selected a thinner humeral intramedullary nail (7 mm) as the main internal fixation device. The fundamental premise for applying a humeral nail to the femur lies in anatomical feasibility. Although the humerus and femur differ in length and curvature, as long tubular bones, their medullary canal structures share similarities in the diaphyseal portion. The key to success in this case was precise preoperative measurement. We confirmed the narrowest diameter of the patient's femoral medullary canal was 4.5 mm, while the selected humeral nail diameter was 7 mm, leaving a safe margin for reaming. By using flexible reamers starting from 5 mm and progressively reaming to 8 mm, we successfully created a channel in a physiologically narrow femur that could accommodate the 7 mm nail while preserving cortical integrity. Intraoperative and postoperative imaging showed a tight fit between the nail and the medullary canal, achieving satisfactory axial and rotational stability even without distal locking.

Buseck et al (4). reported a case of a patient with congenital hip dysplasia who had undergone a proximal femoral varus osteotomy, resulting in a neck-shaft angle of only 98° and a narrow medullary canal (diameter 6 mm), preventing the use of a traditional femoral nail. The authors also chose a humeral intramedullary nail for fixation, pre-bending the nail to adapt to the femoral anterior bow, ultimately achieving stable fracture healing. That study emphasized that for patients with significantly abnormal neck-shaft angles or narrow medullary canals, the humeral nail, due to its smaller diameter and adjustable proximal locking angles, offers a biomechanically superior intramedullary fixation option compared to traditional plates.

Furthermore, Sa-ngasoongsong et al (5). reported the successful application of humeral nails in femoral reconstruction in adolescent patients with osteogenesis imperfecta. Patients with osteogenesis imperfecta often have femoral deformities and narrow medullary canals, sharing similarities with the anatomical characteristics of this case. The authors noted that the humeral nail not only adapts to the narrow canal but also, via a lateral trochanteric entry approach, can avoid iatrogenic injury to the femoral head blood supply while providing better rotational stability than Rush rods.

Of course, the humeral intramedullary nail is not designed for the anatomical morphology of the proximal femur, and its application has certain limitations. For instance, its proximal locking screw angles are typically 90°-100°, which might not be suitable for patients with a normal femoral neck-shaft angle; additionally, distal locking might require longer screws due to the greater width of the distal femur. In this case, the decision to forgo distal locking was based on the fracture type and intraoperative stability assessment, but for more complex fracture types, full locking might be necessary to enhance stability.

To mitigate the risk of nonunion or delayed union resulting from the aforementioned limitations, we enhanced fracture stability intraoperatively through limited open reduction combined with cerclage wiring, thereby compensating for the insufficient angular stability of the humeral intramedullary nail. Postoperative rehabilitation was strictly guided by the principles of individualization, safety, and gradual progression. During the early stage (0–4 weeks), absolute non-weight bearing bed rest was emphasized, supplemented with ankle pump exercises and passive joint mobilization to minimize mechanical stress. In the intermediate stage (5–8 weeks), partial weight bearing was initiated only after radiographic confirmation of callus formation. A gradual transition to full weight bearing was implemented in the later stage (beyond 3 months). This conservative regimen aimed to reduce the risk associated with angular stability deficiency by carefully controlling mechanical load. Follow-up at 30 months confirmed fracture union without complications, validating the effectiveness of this strategy.

## Conclusion

In summary, for special types of subtrochanteric fractures with severe femoral medullary canal stenosis or abnormal neck-shaft angles due to conditions such as poliomyelitis sequelae, osteogenesis imperfecta, or previous osteotomy history, the application of a humeral intramedullary nail is a safe and effective innovative treatment strategy. This approach provides reliable intramedullary fixation and can achieve good fracture healing and functional recovery outcomes. This experience offers new insights for managing similar rare and complex orthopedic problems, but its general applicability still requires further validation with more cases and long-term follow-up.

## Data Availability

The original contributions presented in the study are included in the article/Supplementary Material, further inquiries can be directed to the corresponding author.

## References

[1] Gupta A Saurabh S Trikha T Karpe A Mittal S. Femoral shaft fracture in post-polio syndrome patients: case series from a level-I trauma center and review of literature. Indian J Orthop. (2022) 56(8):1339–46. Published 2022 Jun 29. 10.1007/s43465-022-00683-8 35928657 PMC9283591

[2] Wagh A Shetty V Wagh Y Shekhar S Tandel J. Femur shaft fracture in a polio patient. J Orthop Case Rep. (2022) 12(3):9–12. 10.13107/jocr.2022.v12.i03.2694 36199938 PMC9499046

[3] Mahran M Latif MA Desoky IMI. Causes of failure of subtrochanteric fracture fixation, systematic review and meta-analysis. QJM-An International Journal of Medicine. (2024) 117(Suppl_2):0. 10.1093/qjmed/hcae175.674

[4] Buseck A Koulopoulos M Sullivan MP. The off-label use of a humeral nail to treat a subtrochanteric femur fracture: a case report. Cureus. (2025) 17(1):e77505. Published 2025 January 15. 10.7759/cureus.77505 39958107 PMC11830416

[5] Sa-Ngasoongsong P Saisongcroh T Angsanuntsukh C Woratanarat P Mulpruek P. Using humeral nail for surgical reconstruction of femur in adolescents with osteogenesis imperfecta. World J Orthop. (2017) 8(9):735–40. Published 2017 September 18. 10.5312/wjo.v8.i9.735 28979858 PMC5605360

